# Synthesis and biochemical characterization of EGF receptor in a water-soluble membrane model system

**DOI:** 10.1371/journal.pone.0177761

**Published:** 2017-06-06

**Authors:** Tiffany M. Scharadin, Wei He, Yianni Yiannakou, Alexey A. Tomilov, Matthew Saldana, Gino A. Cortopassi, Kermit L. Carraway, Matthew A. Coleman, Paul T. Henderson

**Affiliations:** 1 University of California Davis School of Medicine, Department of Internal Medicine, Division of Hematology Oncology, Sacramento, California, United States of America; 2 Lawrence Livermore National Laboratory, Livermore, California, United States of America; 3 University of California Davis, Nutrition, Davis, California, United States of America; 4 University of California Davis, School of Veterinary Medicine, Molecular Biosciences, Davis, California, United States of America; 5 University of California Davis School of Medicine, Biochemistry and Molecular Medicine, Sacramento, California, United States of America; 6 University of California Davis Comprehensive Cancer Center, Sacramento, California, United States of America; 7 University of California Davis School of Medicine, Department of Radiation Oncology, Sacramento, California, United States of America; Hungarian Academy of Sciences, HUNGARY

## Abstract

ErbB (Erythroblastic Leukemia Viral Oncogene Homolog) receptor tyrosine kinases are critical for tissue development and maintenance, and frequently become oncogenic when mutated or overexpressed. *In vitro* analysis of ErbB receptor kinases can be difficult because of their large size and poor water solubility. Here we report improved production and assembly of the correctly folded full-length EGF receptor (EGFR) into nanolipoprotein particles (NLPs). NLPs are ~10 nm in diameter discoidal cell membrane mimics composed of apolipoproteins surrounding a lipid bilayer. NLPs containing EGFR were synthesized via incubation of baculovirus-produced recombinant EGFR with apolipoprotein and phosphoplipids under conditions that favor self-assembly. The resulting EGFR-NLPs were the correct size, formed dimers and multimers, had intrinsic autophosphorylation activity, and retained the ability to interact with EGFR-targeted ligands and inhibitors consistent with previously-published *in vitro* binding affinities. We anticipate rapid adoption of EGFR-NLPs for structural studies of full-length receptors and drug screening, as well as for the *in vitro* characterization of ErbB heterodimers and disease-relevant mutants.

## Introduction

The four members of the ErbB family of receptor tyrosine kinases include EGFR or ErbB1/HER1 (human epidermal growth factor receptor), ErbB2/HER2, ErbB3/HER3, ErbB4/HER4. Each ErbB receptor consists of a large extracellular ligand-binding domain, a single transmembrane segment, an intracellular juxtamembrane segment, a tyrosine kinase domain, and a carboxy-terminal tail. These receptors, with the exception of HER2, bind members of the EGF-like growth factor family (e.g. EGF, TGFα, neuregulin) to their extracellular domains leading to a complex conformational change allowing homo- or heterodimerization, autophosphorylation, and activation of pathways that promote cellular proliferation, survival, transformation, migration and invasion [1, 2]. Activation of distinct signaling pathways is determined by the activating ligand and dimerization pair [3–6]. Interestingly, the extracellular domain of HER2 does not bind any known ligands and is frequently observed in the extended conformation allowing for rapid dimerization [7–10]. Recent studies of the ErbB receptor intracellular regions suggest that the kinase domains form an asymmetric dimeric complex in which the amino-terminal end of one receptor binds to the carboxy-terminal end of the other [11–13].

In addition to their physiological functions, the ErbB receptors play roles in the oncogenesis and progression of several cancer types. Most notably, EGFR is overexpressed in 50–80% of non-small cell lung cancers and HER2 and ErbB3 are overexpressed in 25–30% and 63% of breast cancers, respectively [14–20]. Furthermore, the overexpression of multiple members of the ErbB family can enhance tumorigenesis and influence tumor response to ErbB-targeted therapies [21–23]. Two general categories of ErbB-targeted therapies are currently available: monoclonal antibodies that target the extracellular domain (e.g., cetuximab, herceptin) and small molecule tyrosine kinase inhibitors (TKIs; e.g., gefitinib, lapatinib) that block the ATP-binding site on the intracellular domain [24–27]. These treatment strategies have proven effective in various cancer types but ultimately lead to tumor resistance—by activation or mutation of ErbB or parallel signaling pathways—indicating the value of targeting the ErbB receptor but also the need for more specific and efficacious therapeutics [14, 26–38].

Several key details of the mechanism of receptor activation remain to be elucidated, such as definition of the conformational changes that occur within the intracellular domain after ligand binding and the influence of the cell membrane. These important questions are difficult to address as a result of a variety of technical barriers, most notably poor access to large quantities of purified full-length active ErbB receptors in a water-soluble form. To overcome these limitations and provide a more physiological construct, we are incorporating ErbB receptors into NLPs using methods modified from the literature [39, 40]. NLPs are membrane mimetics composed of a phospholipid bilayer surrounded by apolipoproteins ranging in diameter from 8 nm to 17 nm, and have been used to support membrane proteins for studies in native-like membrane conditions [41–46]. Here we synthesized NLPs containing monomeric, dimeric, and multimeric FLAG-tagged EGFR produced as well as purified from insect cells. NLP-associated EGFR is capable of ligand binding, dimerization, autophosphorylation, and inhibition by monoclonal antibodies and tyrosine kinase inhibitors. These results support the utility of EGFR-NLPs as a tool for receptor structural studies and mechanistic studies, and raise the possibility that NLP-receptor complexes can be exploited in screens for more effective therapeutic agents.

## Materials and methods

### Cell culture

Sf9 insect cells were purchased from Life Technologies (Carlsbad, CA). Cells were grown in TMN-FH (HyClone, Logan, UT) supplemented with 10% fetal bovine serum (FBS), 100U/ml penicillin, 100 μg/ml streptomycin, and 0.1% Poloxamer 188 (Corning Mediatech, Manassas, VA) in spinner flasks in a 28°C incubator.

### FLAG-EGFR expression and purification

The FLAG-EGFR plasmid was created in pcDNA3.1 plasmid by inserting the FLAG epitope sequence between a leader sequence peptide and the remainder of the extracellular domain. The FLAG-EGFR sequence was cloned into the flashBACULTRA system to generate an EGFR baculovirus following the manufacturer’s protocol (Oxford Expression Technologies, Oxford, UK). The positive control baculovirus contains a control gene unrelated to the ErbB family, chloramphenical acyltransferase. The FLAG-EGFR baculovirus was incubated with Sf9 insect cells at an MOI of 10 for 48 hours. For Western blot analysis, some cells were treated with 10 ng/ml EGF for 10 minutes prior to collection. The cells were pelleted and frozen at -80°C for storage. The cell pellet was lysed in high triton buffer (TBS plus 1mM EDTA, 1% Triton X-100, and protease/phosphatase inhibitors) and insoluble proteins pelleted. The soluble fraction was incubated with Anti-FLAG M2 Magnetic beads (Sigma-Aldrich, St. Louis, MO) overnight at 4°C. The beads were separated on a magnetic stand and washed twice with high triton buffer and five times with low triton buffer (TBS plus 1mM EDTA, 0.05% Triton X-100, and protease/phosphatase inhibitors). EGFR was eluted from the beads with low triton buffer plus 100 μg/ml FLAG peptide (Sigma-Aldrich) for 1 hr at 4°C.

### Immunocytochemistry

Sf9 insect cells were grown on coverslips in 6-well plates and infected with an MOI of 10 of control or EGFR baculovirus for 48 hours. Cells were washed twice with PBS, fixed in 4% paraformaldehyde, and some cells permeabolized with 100% methanol at -20°C for 10 minutes. Cells were washed in PBS, blocked in 5% BSA, incubated with anti-EGFR antibody followed by anti-rabbit Alexa Flour 555 antibody (Thermo Fisher), mounted with fluoromount G (SouthernBiotech), and imaged.

### NLP assembly and characterization

To form the EGFR-NLPs, we used a protocol modified from the literature [39, 47]. Briefly, 100 μg egg PC (Avanti Polar Lipids, Alabaster, AL) was dried in a glass vial under nitrogen stream. The lipids were solubilized with 10 mM Triton X-100 in PBS with gentle vortexing. To the lipids, 50 μg ApoA1 (Athens Research and Technology, Athens, GA) was added and briefly vortexed. Except where noted, 15 μg EGF (Gold Biotechnology, Olivette, MO) was mixed with approximately 30 μg FLAG-EGFR isolated from insect cells on ice. The EGF/EGFR mixture was added to the lipid/ApoA1 mixture and briefly vortexed and incubated at room temperature for 1–2 hours. To self-assemble the NLPs, the detergent was removed by incubating the assembly mixture with Bio-Beads SM-2 Resin (Bio-Rad, Hercules, CA), which has been pre-hydrated with methanol, on a nutator for 1–2 hours at 4°C. The NLP mixture was removed from the Bio-Beads with a spin column. For egg PC:ApoA1 and EGFR:ApoA1 optimization experiments, assemblies were performed as above with a constant amount of ApoA1 and varying the amount of egg PC or EGFR.

### Size exclusion chromatography (SEC)

Filled (particles containing EGFR) and empty (lacking EGFR) NLPs were separated by a Yarra 3u SEC-4000 column (Phenomenex, Torrance, CA) in SEC buffer (10 mM Tris, 150 mM NaCl, 0.02% sodium azide, pH = 7.5) at 1 ml per minute or a Superdex 200 10/300 column (GE Healthcare, Pittsburgh, PA) at 0.5 ml per minute. SEC protein standards were purchased from Bio-Rad. Appropriate EGFR-NLP fractions were concentrated using 50 kDa cutoff concentrators (Sartorius Stedim, Concord, CA).

### Dynamic light scattering (DLS)

To analyze the NLPs by DLS, a nanotrac particle size analyzer was used in SEC buffer at room temperature (Microtrac, Montgomeryville, PA). Three 30-second measurements were taken and averaged per sample.

### Electrophoretic and immunoblotting analysis

Samples for Coomassie stain and immunoblotting were electrophoresed on 4–12% Bis-Tris gels (Life Technologies) with or without β-mercaptoethanol (β-ME) and heat. For Coomassie, the gel was incubated with Coomassie Brilliant Blue R250 (Thermo Fisher Scientific, Waltham, MA) and scanned. For immunoblot, the proteins were transferred to a nitrocellulose membrane using the iBlot Dry Blotting System (Life Technologies/Invitrogen) and immunostained using the indicated antibodies. For the dotblot, 3 μl of each sample were pipetted onto a nitrocellulose membrane and immunostained with the indicated antibodies. For native gel electrophoresis, samples were separated on a 4–16% Bis-Tris gel using a Novex NativePAGE Bis-Tris gel system (Thermo Fisher Scientific), transferred to PVDF, and immunostained with indicated antibodies. EGFR (1005) antibody was purchased from Santa Cruz Biotechnology (Santa Cruz, CA). FLAG M2 antibody was purchased from Sigma-Aldrich. pY20 antibody was purchased from BD Biosciences (San Jose, CA). pY4G10 antibody was purchased from EMD Millipore (Billerica, MA). ApoA1 (5F4), pEGFR (Tyr1068), anti-mouse HRP, and anti-rabbit HRP antibodies were purchased from Cell Signaling Technology (Danvers, MA). Band densitometry was determined using ImageJ software and normalized to the amount of ApoA1 protein.

### ELISA analysis

EGFR concentrations in the assembly mixture and in EGFR-NLP preparations were determined using the human total EGFR duoset IC kit from R&D Systems (Minneapolis, MN). Concentrations were calculated using the four parameter logistic curve at myassays.com.

### Carbonate extraction

Carbonate extraction was performed as previously described [48]. Briefly, EGFR-NLPs were diluted 1:10 in PBS (control) or 0.1 M sodium carbonate (Na_2_CO_3_). Samples were incubated on ice for 30 minutes followed by centrifugation at 14,000 x g for 10 minutes. The supernatant (S) was removed as the soluble fraction and the pellet (P) was resuspended in an equal volume of the same buffer. All samples were separated on denaturing SDS-PAGE gels and analyzed by Western blotting with anti-EGFR antibody.

### Ligand binding

EGFR binding to EGF was determined using BLI implemented by the Octet RED384 System (Pall ForteBio LLC, Menlo Park, CA). EGF purchased from Gold Biotechnology was biotinylated using the EZ-link sulfo-NH2-biotin kit from Thermo Fisher Scientific according to the manufacturer’s protocol. Biotinylated EGF (10 μg/ml) was loaded onto sets of Octet RED384 SA biosensors (Pall ForteBio LLC) followed by blocking of non-occupied streptavidin residues on the biosensors with 200 μM biotin. After stable baseline was achieved, the sensors were tested against a 1.5-fold titration series of empty NLP or EGFR-NLPs prepared in BLI Kinetic buffer (Pall ForteBio LLC) + 0.1% BSA to molar concentrations between 1.5 and 670 nM for 2000 s (association) followed by buffer without NLPs for 2000 s (dissociation). A set of biosensors loaded with biotinylated GFP instead of EGF was used as a negative control to compensate for possible signal drift due to buffer or non-specific binding of NLPs to the sensors. Plots of association responses (nm) from 1990–1995 s were created against log concentration [M] of empty or EGFR-NLPs and EC_50_ and Hill Slopes were calculated using a sigmoidal dose response fitting with variable slope in GraphPad Prism. To determine the inhibition of EGFR-NLP to EGF binding, 100 nM EGFR-NLPs were incubated with 1 μM unlabeled EGF or a 1.5-fold titration series of cetuximab concentrations between 0.2 and 100 nM during the assay. Cetuximab was generously provided by the UC Davis Comprehensive Cancer Center.

### Kinase assay

EGFR-NLPs were assembled in the presence of EGF. To remove any phosphorylation, the NLPs were incubated with 1U calf intestinal phosphatase (CIP) in CutSmart buffer (NEB, Ipswich, MA) for 1 hour at room temperature. Following dephosphorylation, the phosphatase was inhibited with sodium orthovanadate. To promote auto phosphorylation, the EGFR-NLPs were incubated with kinase buffer (15 mM MgCl_2_, 2 mM DTT, 0.3 mM ATP) with and without 20 ng/ml EGF and 1 μM tyrosine kinase inhibitor (mubritinib, afatinib, canertinib, or lapatinib) for 2 minutes on ice. The tyrosine kinase inhibitors were purchased from LC laboratories (Woburn, MA). Phosphorylation was detected by immunoblot.

### Statistical analysis

Statistical analyses were carried out using GraphPad Prism.

## Results

### Production, assembly, and initial characterization of EGFR-NLPs

**Fig 1:**
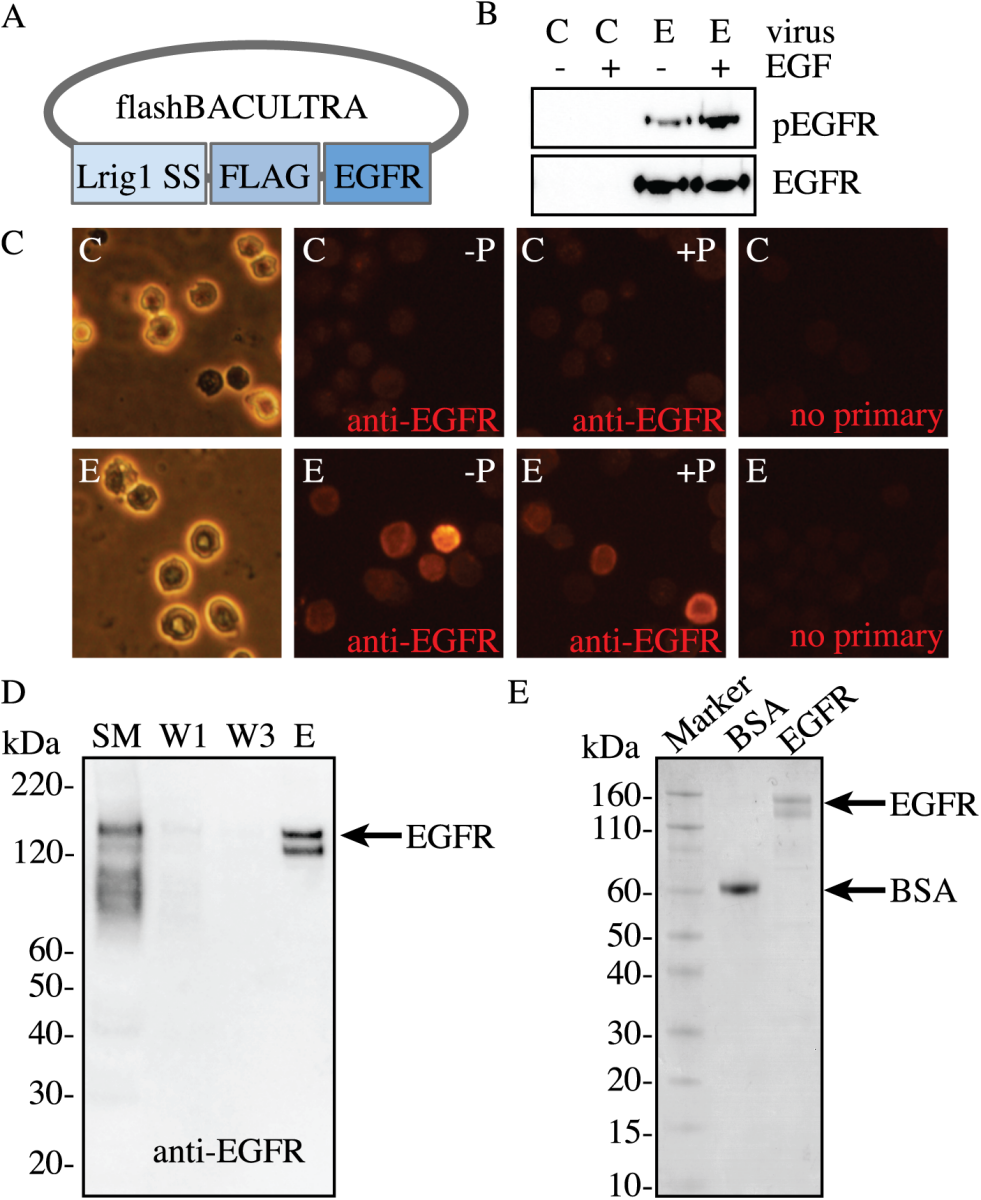
Baculovirus-expressing EGFR production. *A*, Map of the EGFR baculovirus using the flashBACULTRA system with a FLAG epitope placed between the leader sequence peptide (Lrig1 SS) and the full-length mammalian EGFR sequence (EGFR). *B*, Sf9 insect cells were infected with a MOI of 10 of control baculovirus (C) or EGFR baculovirus (E) for 48 hours followed by collection of total cell lysate and Western Blotting to detect EGFR expression. A set of these cells was treated with 10 ng/ml EGF in order to detect ligand-induced receptor phosphorylation. *C*, Sf9 insect cells were seeded on glass coverslips and infected with an MOI of 10 of control (C) or EGFR (E) baculovirus for 48 hours. Cells were fixed in 4% paraformaldehyde followed by immunostaining with anti-EGFR antibody (in red) with (+P) or without (-P) permeabilization in 100% methanol. The brightfield image shows cell morphology. Cells incubated with secondary antibody in the absence of EGFR antibody (no primary) were used as a negative control. *D*, FLAG-EGFR is readily purified from Sf9 insect cells as shown by a representative image of anti-EGFR immunoblot of FLAG EGFR purification and *E*, a Coomassie-stained SDS-PAGE gel. Bovine serum albumin (BSA) was loaded as a protein of known concentration. Total insect cell lysate starting material (SM), purification washes 1, 3 (W1, W3), and elution (E) were loaded to show efficiency of the FLAG-purification.

An EGFR baculovirus with a membrane signaling sequence and FLAG-tag was made using the flashBACUltra sytem (Fig 1*A*). Insect cells were infected with a multiplicity of infection (MOI) of 10 of control or EGFR baculovirus for 48 hours with and without additional EGF followed by detection of total and phosphorylated EGFR by Western Blot (Fig 1*B*). A basal amount of phosphorylated EGFR is observed due to EGF being present in the fetal bovine serum in the cell culture medium. We next confirmed that the EGFR was expressed on the cell membrane by immunocytochemistry with and without cell permeabolization (Fig 1*C*). For purification, EGFR was isolated from the insect cells after a 48-hour infection with an MOI of 10 of EGFR baculovirus via FLAG-purification from total cell lysates, which resulted in a band whose migration on an SDS-PAGE gel was consistent with full-length EGFR as shown by immunoblotting and Coomassie (Fig 1*D and* 1*E*).

**Fig 2:**
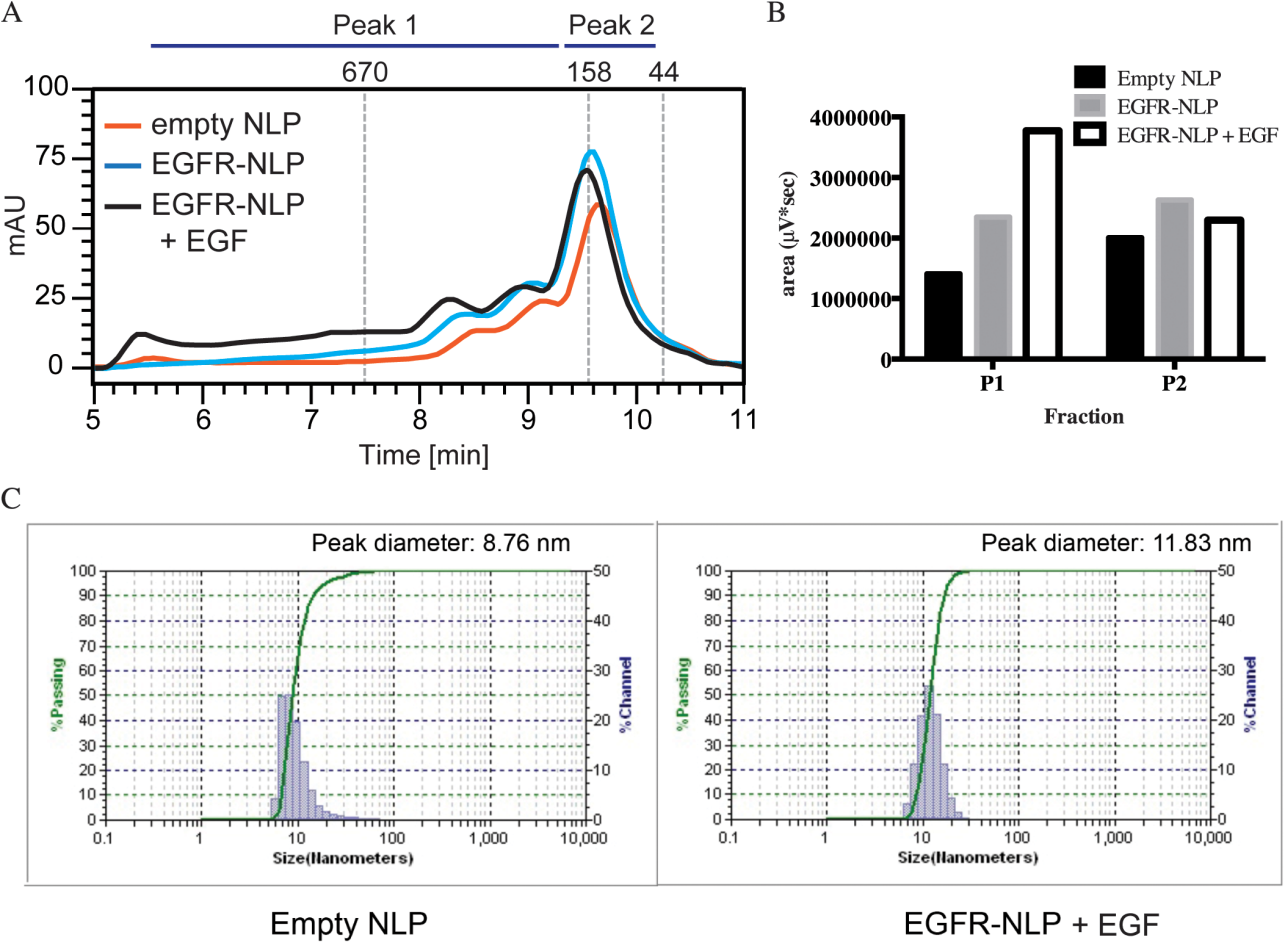
Characterization of EGFR-NLP size and shape. *A*, Following NLP self-assembly, the specified NLPs were separated by a Yarra 3u SEC-4000 column and typical traces are shown. Empty NLPs elute between 9.5–10.5 minutes (P2) with EGFR-NLPs eluting earlier at 5.5–9.5 minutes (P1). Numbers above indicate molecular mass (in kDa) of known protein standards. *B*, Quantification of area under the curve of the P1 and P2 SEC peaks in μV*sec from a typical SEC trace. *C*, Empty and EGFR-NLPs were analyzed by DLS to determine NLP diameter.

**Fig 3:**
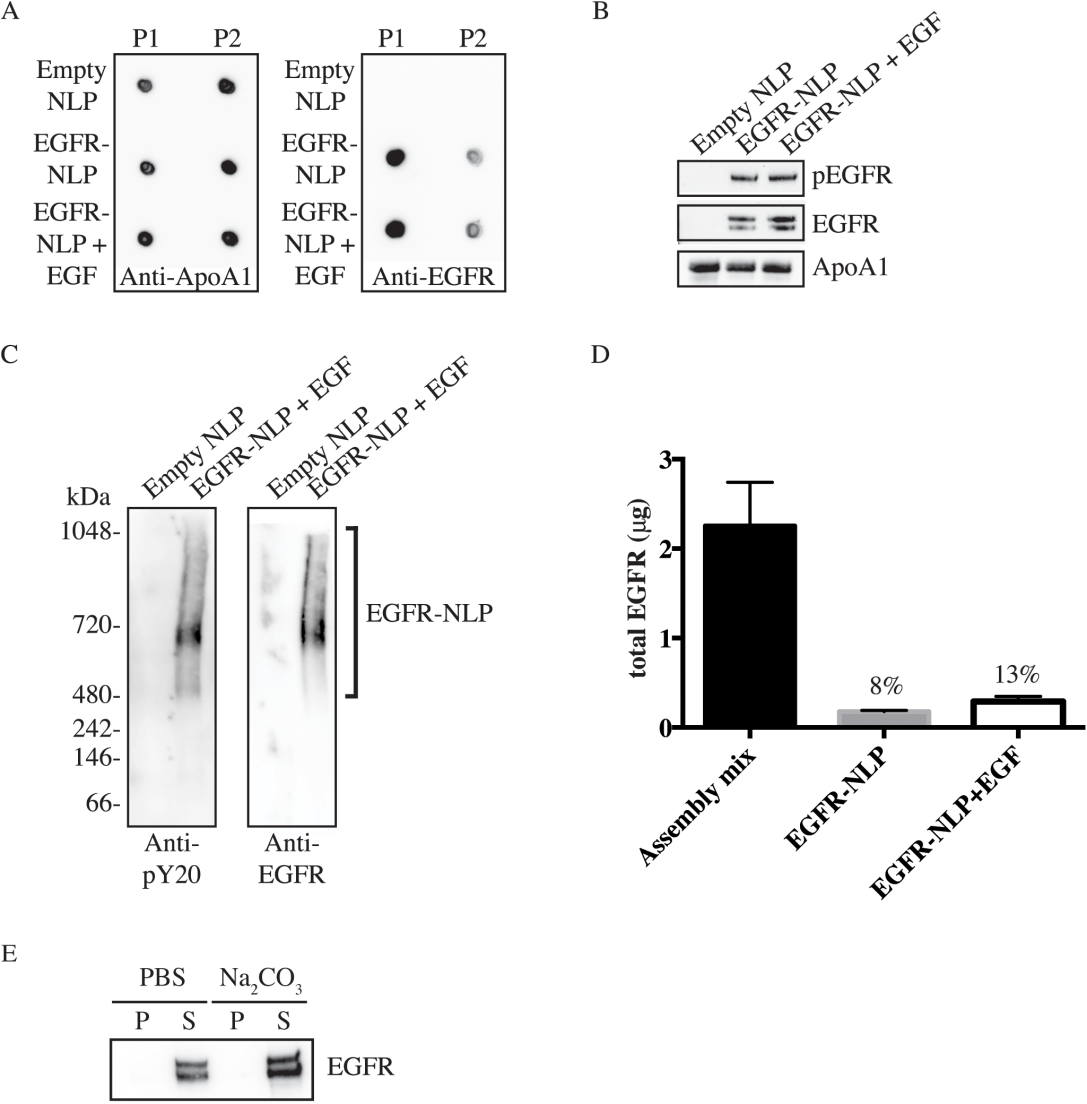
EGFR incorporation in self-assembled NLPs. *A*, P1 and P2 SEC fractions were collected and spotted onto nitrocellulose membrane followed by immunoblotting with anti-ApoA1 and anti-EGFR antibodies showing that the majority of EGFR is incorporated into the higher molecular weight NLPs of the P1 fraction. *B*, Indicated NLPs were separated by denaturing SDS-PAGE and analyzed with anti-pEGFR, anti-EGFR, and anti-ApoA1 antibodies. Numbers indicate band intensity relative to EGFR-NLP normalized to ApoA1 signal. *C*, Purified Empty and EGFR-NLPs were separated by NativePAGE gels and immunostained with anti-pY20 and anti-EGFR antibodies showing EGFR expression in the higher molecular weight ranges above 480 kDa. *D*, Bar graph of determination of EGFR insertion into NLP. Total amount of EGFR in the NLP assembly mixture and purified EGFR-NLPs were determined by ELISA and EGFR insertion rate indicated. The values represent the mean ± standard error of the mean (SEM) for two technical replicates. *E*, EGFR-NLPs were extracted with PBS (control) or sodium carbonate (Na_2_CO_3_), centrifuged to separate the supernatant containing NLPs (S) and pellet containing insoluble free protein (P), separated by denaturing SDS-PAGE, and analyzed with anti-EGFR antibody showing that EGFR is not susceptible to carbonate extraction.

We next assembled EGFR-NLPs by combining purified EGFR with or without its ligand, EGF, Apolipoprotein A1 (ApoA1), egg phosphatidylcholine (egg PC), and triton X-100. This was followed by detergent removal using Bio-Beads. This assembly results in a mixture of receptor-free “empty” NLPs, EGFR-filled NLPs, and unincorporated EGFR which have large differences in molecular weight that can be separated via size exclusion chromatography (SEC) by comparing the elution times to a set of protein standards (Fig 2*A*). Empty control NLPs were formed in the absence of EGFR and EGF. Empty NLPs typically elute near the 158 kDa standard (9.5–10.5 min), which we called peak 2 (P2), while the higher molecular weight EGFR-NLPs eluted earlier and are denoted as peak 1 (P1) (5.5–9.5 min). We were able to quantify the area under the SEC curve for each assembly mixture. In a typical reaction, the fraction showing the highest amount of protein in P1 was in the EGFR-NLP+EGF assembly (3771481 μV*sec), followed by the EGFR-NLP (2344794 μV*sec) and empty NLP (1409463 μV*sec) assemblies, while the amount of protein in the P2 fraction was similar (2294813 vs. 2629933 vs. 2003750, respectively) (Fig 2*B*). Egg PC:ApoA1 and EGFR:ApoA1 ratios were optimized as displayed in S1*A and* S1*B* Fig. NLP assemblies with a broad peak can be rerun to obtain NLPs of a uniform size (S1*C* Fig). We next assessed the size distribution of the NLPs by dynamic light scattering (DLS). Both empty and EGFR-NLPs+EGF had relatively small size distributions of 5–70 nm or 6–30 nm, respectively. However, the peak SEC fraction of material was slightly larger for the EGFR-NLPs (11.83 nm) than the empty NLPs (8.76 nm) (Fig 2*C*), which is consistent with incorporation of membrane proteins [40]. To verify the protein composition in the SEC peaks, the P1 and P2 factions were collected, concentrated, and spotted on nitrocellulose membrane for immunoblotting (Fig 3*A*). As expected, ApoA1 was present in both peaks of all assemblies, but the majority of EGFR was found in P1 for the assemblies containing EGFR. We next separated the NLP assemblies by SDS-PAGE and immunostained with pEGFR, EGFR, and ApoA1 antibodies to demonstrate that full-length EGFR is the produced at the expected molecular weight of 175 kDa (Fig 3B). The SDS-PAGE analysis also illustrates that the EGFR and the NLP are associated. Furthermore, we separated the NLP assemblies via NativePage gels (Fig 3*C*). For the native gel comparison, we observed the EGFR-NLPs spanned a large range of molecular weights above 480 kDa, indicating a range in the number of EGF receptors associated per NLP with a predominant band at the expected molecular weight of approximately 600 kDa for an EGFR homodimer NLP. These results are similar to what was observed by SEC separation. Interestingly, the EGFR-NLPs along the entire range appeared to be phosphorylated as determined by immunoblot.

To quantitate the amount of EGFR that was incorporated into our NLPs, we performed an ELISA on total EGFR protein levels in the NLP assembly mixture and purified EGFR-NLPs (Fig 3*D*). We calculated the percentage of EGFR that was incorporated into NLPs by dividing the amount of EGFR in each of the NLP groups by the amount of EGFR in the assembly mixture. The incorporation rate varied slightly with each assembly (8–20%), but typically was near the previously reported value of approximately 10% [39]. However, we observed a higher level of EGFR incorporation in the presence of EGF (8 vs. 13%), although it did not reach statistical significance. This was also consistent with the SEC area under the curve measurements. We further confirmed that EGFR is incorporated into the NLPs by performing a carbonate extraction experiment. Peripherally bound proteins can be extracted by carbonate while integral membrane proteins are not. We observed that no EGFR was extracted from the EGFR-NLPs by carbonate indicating that EGFR is integrated into the NLP (Fig 3*E*).

### NLP-incorporated EGFR is capable of EGF binding

**Fig 4:**
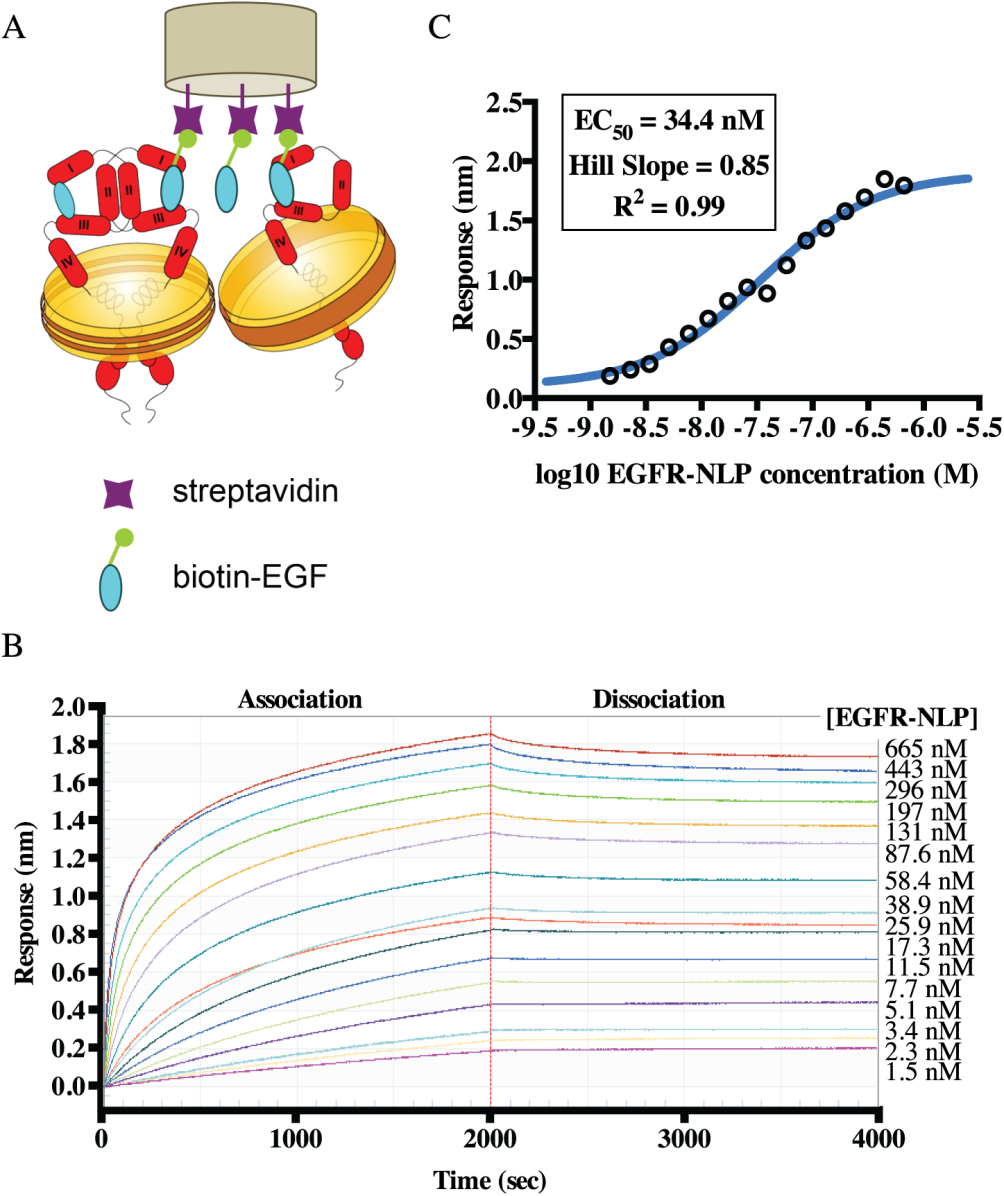
NLP-associated EGFR is capable of binding its ligand, EGF. *A*, Schematic of the OctetRed assay used to determine EGFR ligand binding. Streptavidin biosensors were loaded with biotinylated EGF and EGFR-NLP association and dissociation with the sensor was quantitated via BLI. *B*, Association (0–2000 s) and dissociation (2000–4000 s) curves of EGFR-NLPs binding to biotinylated EGF on the sensors. EGFR-NLP concentrations were titrated by a 1.5-fold dilution to the concentrations displayed to the right of the graph. Curves are normalized by subtracting the 0 μM EGFR-NLP curve. *C*, Log plot of EGFR-NLP binding response (nm) to biotinylated EGF. Plot shows mean of two technical duplicates and are representative of results from three biological repeats.

We next determined the ability of EGFR-NLPs to bind EGF, the natural ligand of EGFR suggesting that the extracellular domain is properly folded. To quantitate EGF binding, we loaded biotinylated EGF onto streptavidin biosensors of an Octet RED384 instrument and measured NLP binding using Bio-Layer Interferometry (BLI) as depicted in Fig 4*A*. The sensors were incubated with 1.5–670 nM EGFR-NLP for 2000 s to determine association followed by 2000 s in buffer to determine dissociation (Fig 4*B*). Using the association response from the range of EGFR-NLP concentrations, we determined the EC_50_ (effective dose, 50%) for EGFR-NLP to EGF binding of 34.4 nM (95% confidence interval (CI) = 2.98–3.98 nM) (Fig 4*C*). The Hill Slope of this interaction, 0.85, was approaching 1 indicating that one EGF binds to one EGFR molecule. In contrast, we did not observe binding of empty NLPs to biotin-EGF or binding of NLPs to the biotin-GFP control.

### NLPs containing EGFR homodimers are sensitive to inhibition by EGFR-targeted therapies

**Fig 5:**
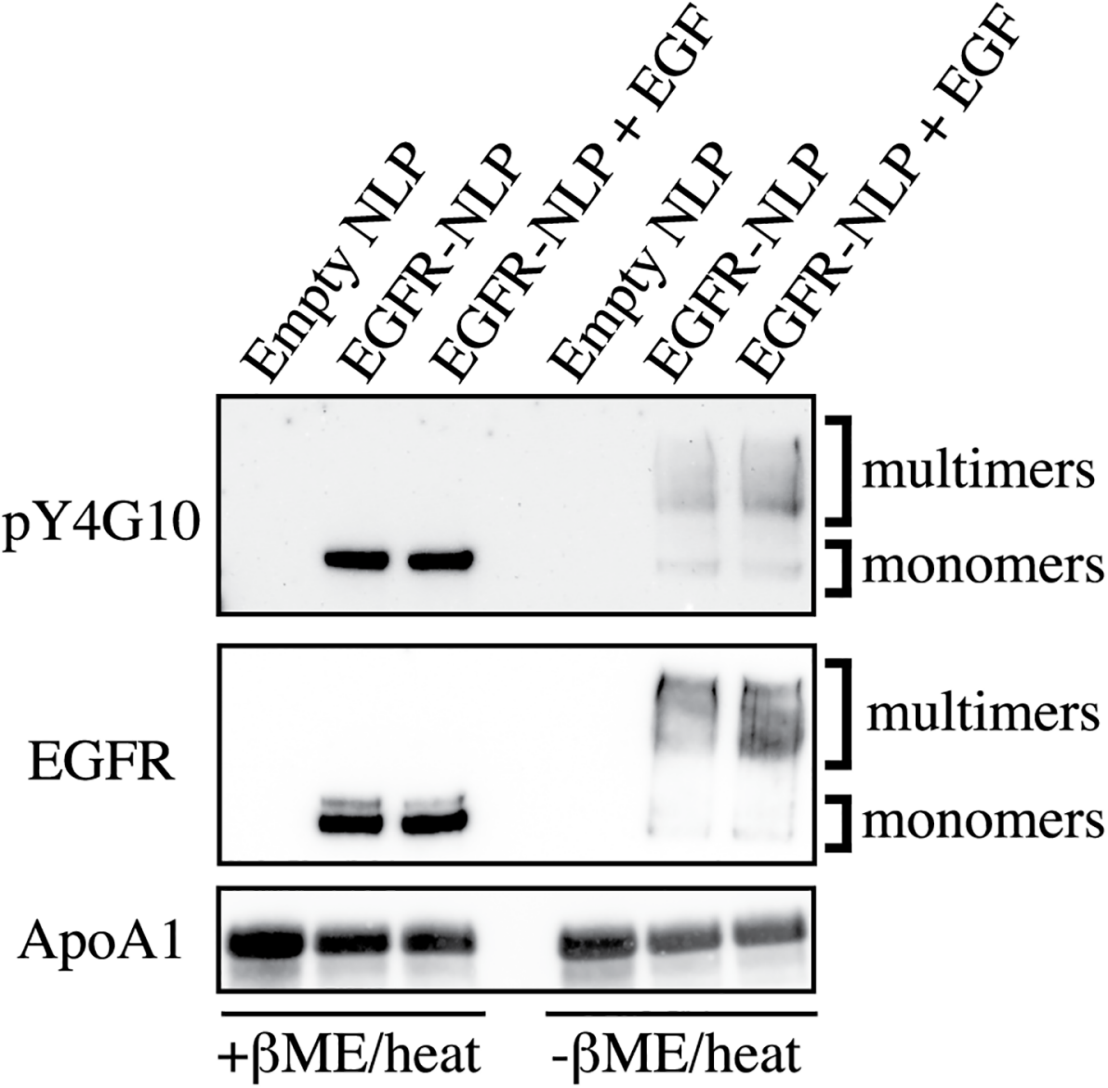
NLP assemblies contain EGFR monomers, dimers, and multimers. Empty and EGFR-NLPs were separated by SDS-PAGE with (+β-ME/heat) and without denaturing conditions (-β-ME/heat) to observe EGFR multimers and analyzed by immunoblotting with anti-pY4G10, anti-EGFR, and anti-ApoA1 antibodies. Similar results were observed in each of three biological replicates. Numbers indicate band intensity relative to EGFR-NLPs normalized to ApoA1 signal.

Biologically, the presence of EGF often increases the formation of EGFR dimers, so we hypothesized that adding EGF to the NLP assembly mixture would produce a higher yield of EGFR-NLPs containing homodimers as well as multimers compared to the assembly mixtures made without EGF supplementation. In order to determine the extent of EGFR complex formation, we separated the empty and EGFR-NLPs on a single PAGE gel with and without reducing agent and heat (Fig 5). With reducing conditions, both NLPs and EGFR complexes are separated, and without reducing conditions, NLPs separate but EGFR complexes remain intact. Under reducing conditions (left side of blot), we observed higher amounts of EGFR and pEGFR in assemblies containing EGF. Without heat and reducing agents (right side of blot), we observed a wide smear of EGFR and pEGFR larger than the monomer band that likely indicated EGFR dimers and/or larger multimers. As hypothesized, the addition of EGF to the assembly mixture increased the yield of EGFR and pEGFR incorporated into the NLPs.

**Fig 6:**
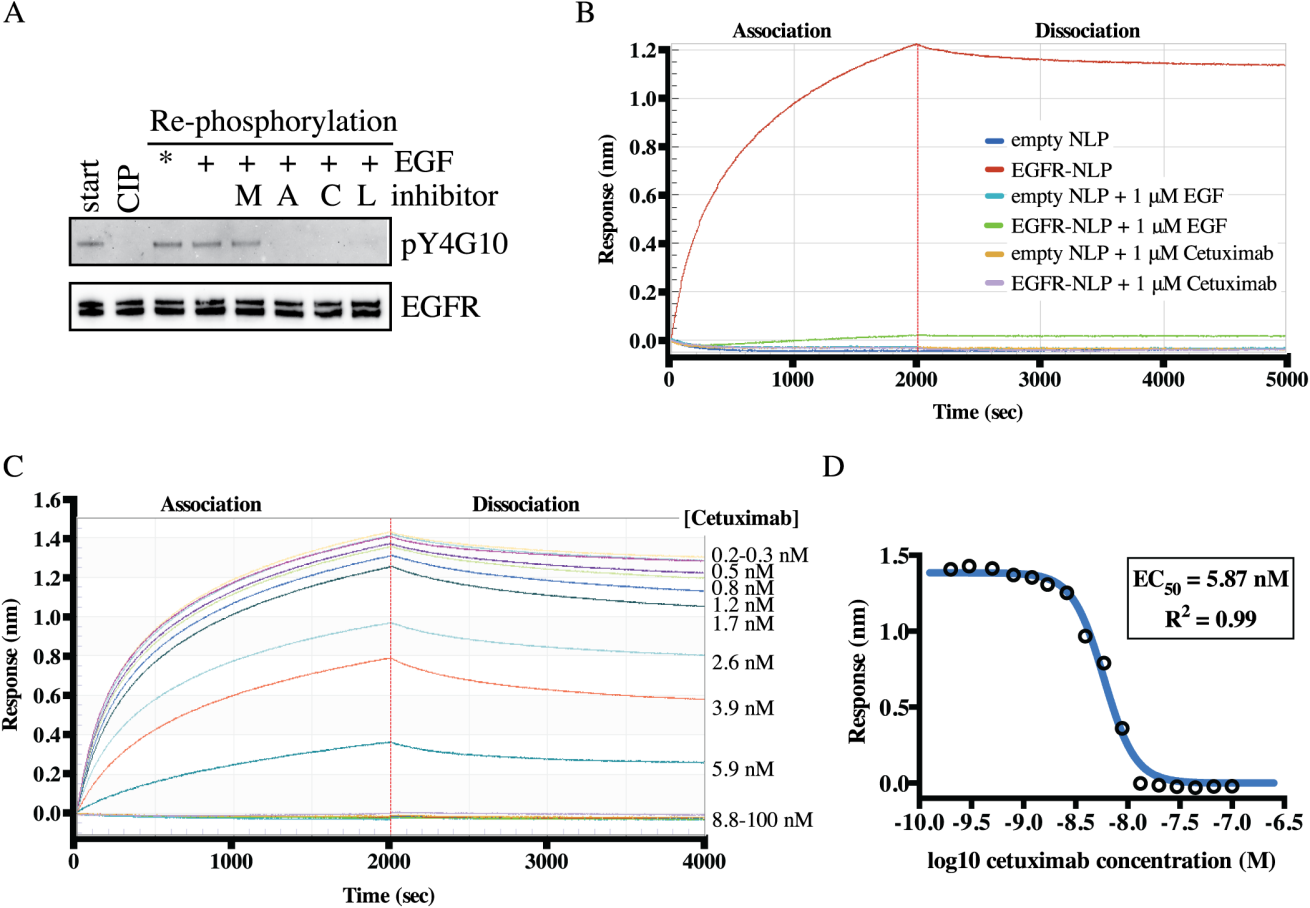
NLP-associated EGFR interacts with EGFR-targeted therapeutic agents. *A*, NLP incorporated EGFR was dephosphorylated via phosphatase treatment (CIP) then allowed to autophosphorylate in the absence or presence of indicated tyrosine kinase inhibitors (M, mubritinib; A, afatinib; C, canertinib; L, lapatinib) followed by immunoblotting with anti-pY4G10 and anti-EGFR antibodies. Similar results were observed in each of three biological replicates. *Autophosphorylation is observed without additional EGF due to EGF being present in the fetal bovine serum and in the initial assembly mixture. *B*, Association and dissociation of 100 nM of empty or EGFR-NLP to biotinylated EGF on the sensors with and without 1 μM unlabeled EGF or cetuximab. Data points indicate the mean of two technical duplicates and are representative of results from two biological replicates. *C*, Association and dissociation curves of 100 nM EGFR-NLPs binding to biotinylated EGF on the sensors in the presence of increasing levels of Cetuximab. Cetuximab concentrations were titrated by a 1.5-fold dilution to the concentrations displayed to the right of the graph. Curves were normalized by subtracting the 0 μM Cetuximab curve. *D*, Log plot of cetuximab inhibiting the binding of EGFR-NLPs to biotinylated EGF on the sensors. Data points indicate the mean of two technical duplicates and are representative of results from two biological replicates.

We next determined if the NLP-incorporated EGFR is enzymatically functional suggesting a properly folded intracellular domain by analyzing EGFR autophosphorylation and active site inhibition (Fig 6*A*). EGFR-NLP assemblies can contain a basal amount of phosphorylated EGFR because EGF is present in the FBS of the Sf9 culture media and is added to the NLP assembly mixture (start). To test if the EGFR was able to autophosphorylate after purification and incorporation into NLPs, we removed the phosphates from the EGFR-NLPs using calf intestinal phosphatase (CIP) and then incubated the EGFR-NLPs with kinase buffer and ATP with or without kinase inhibitors. The EGFR-NLPs were able to autophosphorylate in the kinase buffer with and without additional EGF. This was not surprising because the EGFR-NLPs were assembled in the presence of EGF thus additional EGF does not increase phosphorylation levels. EGFR was also able to autophosphorylate in the presence of the HER2-specific inhibitor (mubritinib), but not in the presence of an EGFR-specific inhibitor (afatinib), a pan-ErbB inhibitor (canertinib), or an EGFR/HER2 inhibitor (lapatinib). These findings indicate that the EGFR in EGFR-NLPs was capable of kinase activity and can be used to screen inhibitors of autophosphorylation.

To determine the ability of monoclonal antibodies to block EGFR-NLPs binding to EGF, we again used the BLI system depicted in Fig 4*A*. EGFR-NLPs in the absence of inhibitor were able to bind to EGF, but the addition of 1 μM of unlabeled EGF or the EGFR monoclonal antibody, cetuximab, to the mixture completely inhibited EGFR-NLP binding to EGF (Fig 6*B*). Neither empty NLPs nor EGFR-NLPs bound to the biotin-GFP control. We then incubated 100 nM EGFR-NLPs with 0.2–100 nM cetuximab and determined association and dissociation for 2000 s each (Fig 6*C*). Using the association response from the range of cetuximab concentrations, we determined an EC_50_ of 5.87 nM (95% CI = 5.33–6.47 nM) (Fig 6*D*). These findings indicate that NLP-associated EGFR was able to bind to its natural ligand, EGF, forms dimers and multimers, and together with the kinase data strongly suggests that the full-length EGFR is functional and correctly folded.

## Discussion

Here we present the production as well as assembly of full-length EGFR monomers, homodimers, and multimers into NLPs at a 10–20% incorporation rate, the characterization of EGFR-NLPs size and function, and studies of their inhibition by EGFR-targeted drugs. Because EGFR is a membrane receptor and has low water solubility, several groups have reconstituted EGFR into membrane mimetics such as bicelles, proteoliposomes, or NLPs to achieve a more native and soluble form of EGFR for biochemical studies [39, 49–54]. Our group chose to incorporate EGFR into NLPs because NLPs negate the issue of orientation of the EGFR compared to proteoliposomes, a protocol has already been shown to yield acceptable EGFR incorporation with stable receptor and kinase activity for at least 24 hours, and our extensive group expertise in assembling NLPs [39]. Previous studies that incorporated EGFR into NLPs used EGFR that was isolated from mammalian cells or generated through a cell-free expression system [39, 49, 50]. In contrast, we have developed an EGFR baculovirus expression system for large scale production in insect cell culture, which provides us with a more robust supply of protein (up to 0.8 mg purified full-length EGFR per liter of insect cell culture). Successful production of full-length EGFR recombinant protein via baculovirus has previously been reported and used for biochemical receptor studies [55–57]. Furthermore, we combined aspects from published assembly protocols to produce EGFR-NLPs in as few as five hours compared to more than 16 hours with similar incorporation rates [39, 47]. The increased solubility and stability of EGFR in an NLP in combination with enhanced protein production and decreased assembly time makes NLP systems ideal for the studies presented here and future screening and structural studies.

We addressed an important aspect that was not previously clarified: is the EGFR associated with NLPs in the monomeric or multimeric state? EGFR-NLPs separated by SEC show protein eluting along a large size range earlier than the empty NLP peak around 158 kDa, indicating there is a mixture of EGFR monomers, dimers, and multimers in these assemblies. When EGF is added to the EGFR-NLP assembly mixture, a higher yield of protein is observed at the higher molecular weight regions, which would include the expected size for NLPs containing EGFR homodimers and multimers. Using a NativePAGE gel, we observe a large band around the expected size of an NLP containing an EGFR homodimer, with the total EGFR-NLPs spanning a larger size range. We ran the EGFR-NLPs on an SDS-PAGE gel without β-ME or heat, which removed the EGFR from the NLP but did not disturb dimer and multimer formations. We observed a small band consistent with EGFR monomers and a large band at a higher molecular weight suggesting that the majority of the NLPs contain EGFR homodimers or multimers. Further, the addition of EGF to the assembly mixture increased the amount of protein in the higher molecular weight band, supporting our hypothesis that the addition of EGF to the mixture enhanced EGFR homodimer and multimer formation. Similar to our findings, the Simons group observed EGFR dimer formation in proteoliposomes in the absence of EGF that increased when EGF was added [51]. They also found evidence that the lipid environment plays a role in dimer formation as the inclusion of ganglioside GM3 reduced dimer formation. Further studies on the role of lipid and membrane components in EGFR dimerization can easily be compared using the proteoliposome or NLP platforms.

We confirmed that our EGFR-NLPs are capable of binding EGF, the natural ligand of EGFR, and determined an EC_50_ of 34.4 nM and Hill Slope of 0.85. This value is slightly higher than the EC_50_ for cells binding to EGF (0.13–11.4 nM) but comparable to that observed for soluble EGFR or proteoliposome-incorporated EGFR binding to EGF on a similar system, Biacore (1.2–175 nM) [51, 58–60]. NLP-associated EGFR would be expected to show binding more similar to that of the protein bound to the cell membrane as it is in a more native confirmation than detergent-solubilized EGFR. Additionally, we were able to completely inhibit the EGFR-NLPs from binding EGF with unlabeled EGF or the EGFR-targeting monoclonal antibody Cetuximab. We observed an EC_50_ of 5.87 nM for Cetuximab inhibition of EGF binding, which is slightly higher but relative to previously reported EC_50_ values of 0.1–5.3 nM in both cell-based and soluble protein assays [61–64].

Finally, we addressed whether our EGFR-NLPs contain active kinases that can be inhibited by EGFR-targeted therapies. Previous studies have shown that both proteoliposome and NLP-incorporated EGFR is capable of phosphorylating itself or an external substrate, and its kinase activity can be inhibited [39, 49–53]. Unlike NLPs, the use of proteoliposomes requires permeabolization for ATP to gain access to the kinase domain. Similar to dimer formation, studies report a basal level of phosphorylated EGFR that increases with the addition of EGF, particularly with higher concentrations of EGFR [52, 53]. Interestingly, the addition of the ganglioside GM3 or decrease of the temperature and thus membrane fluidity were able to reduce phosphorylation of EGFR in proteoliposomes [51, 53]. We also observed that EGFR-NLPs are capable of autophosphorylation which can be enhanced with EGF or inhibited by the ErbB or EGFR-specific tyrosine kinase inhibitors, afatanib, canertinib, and lapatinib, but not the ErbB2-specific inhibitor, mubritinib. This demonstrates the specificity of our baculovirus-produced EGFR as well as the capabilities of membrane mimetics to study inhibition of membrane receptor kinase activity by therapeutic agents and the influence of the membrane composition that supports the use of NLPs for rapidly screening potential new EGFR-targeted therapies. However, conformational heterogeneity of the receptor in the analyzed samples cannot be excluded. It is possible that only a minor fraction of the EGFR-NLPs might have been functionally active. Future work will focus on improving the synthesis and purification to high homogeneity, which will not only enable more quantitative biochemical analysis, but also likely facilitate structural characterization of full length receptors—a long sought goal.

In conclusion, we have improved the production and assembly time of EGFR-NLPs utilizing a robust insect cell baculovirus system. We demonstrated that our EGFR-NLPs are functional for oligomerization and recognition by both native ligands and conformational antibodies suggesting that they are properly folded. This study also demonstrates how these particles can be used as reagents for screening potential EGFR-targeted therapies. In the future, we plan to use this system for structural studies on the full-length EGFR protein using cryoEM and small angle X-ray scattering (SAXS), which have previously only been attainable on truncated forms of EGFR. We also plan to adapt this system to study EGFR heterodimers and disease-relevant mutants.

## Supporting information

S1 Fig Optimization of EGFR-NLP assemblies. *A*, Empty NLPs were assembled with increasing amounts of egg PC at listed ratios followed by separation by Superdex 200 SEC column and typical traces are shown. Empty NLPs elute between 22–26 minutes. *B*, EGFR-NLPs were assembled with increasing amounts of EGFR at listed ratios followed by separation by Superdex 200 SEC column and typical traces are shown. Empty NLPs elute between 22–26 minutes with EGFR-NLPs eluting earlier at 16–22 minutes. Triton-solubilized EGFR was eluted between 36–38 minutes. *C*, An empty NLP assembly was separated by Superdex 200 SEC column (top). To obtain a more uniform NLP size, center fractions were collected, concentrated, and reseperated by SEC column (bottom).
(TIF)
