# Supplementary figures and images for: Synthesis and biochemical characterization of EGF receptor in a water-soluble membrane model system
Source: PLoS One. 2017 Jun 6;12(6):e0177761. doi: 10.1371/journal.pone.0177761 (PMC5460842; doi:10.1371/journal.pone.0177761)

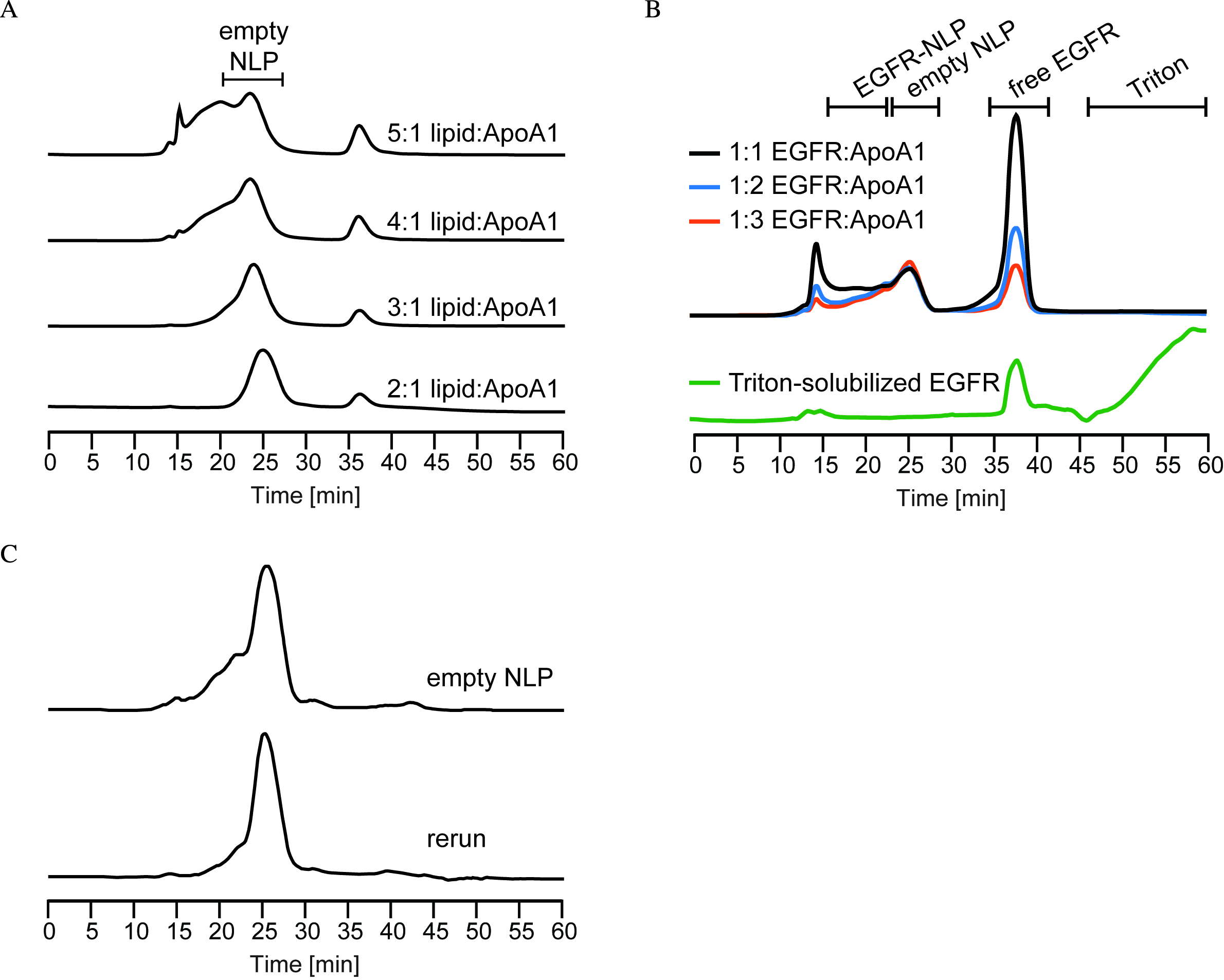

Supplement: S1 Fig — A, Empty NLPs were assembled with increasing amounts of egg PC at listed ratios followed by separation by Superdex 200 SEC column and typical traces are shown. Empty NLPs elute between 22–26 minutes. B, EGFR-NLPs were assembled with increasing amounts of EGFR at listed ratios followed by separation by Superdex 200 SEC column and typical traces are shown. Empty NLPs elute between 22–26 minutes with EGFR-NLPs eluting earlier at 16–22 minutes. Triton-solubilized EGFR was eluted between 36–38 minutes. C, An empty NLP assembly was separated by Superdex 200 SEC column (top). To obtain a more uniform NLP size, center fractions were collected, concentrated, and reseperated by SEC column (bottom). (TIF) [file pone.0177761.s001.tif]
